# Destination Transplant: Protocol for a Parallel-group Randomized Trial of an Educational Intervention to Increase Kidney Transplant Among Black People on the Transplant Waiting List

**DOI:** 10.1097/TXD.0000000000001136

**Published:** 2021-03-16

**Authors:** Francis L. Weng, LaShara A. Davis, Pamela A. Ohman-Strickland, Amy D. Waterman

**Affiliations:** 1 Renal and Pancreas Transplant Division, Saint Barnabas Medical Center, Livingston, NJ.; 2 Rutgers University School of Public Health, Piscataway, NJ.; 3 Division of Liberal Arts and Social Sciences, DeSales University, Center Valley, PA.; 4 Division of Nephrology, David Geffen School of Medicine, University of California, Los Angeles, Los Angeles, CA.; 5 Terasaki Institute for Biomedical Innovation, Los Angeles, CA.

## Abstract

For most patients with kidney failure, living donor kidney transplant (LDKT) is their best treatment option. Compared with White people, Black people are more likely to have kidney failure but less likely to receive LDKTs. In this study, the investigators will test an educational intervention, Destination Transplant, designed to reduce this disparity, among Black people already listed for kidney transplant.

**Methods.:**

The investigators will conduct a parallel group, 2-arm randomized clinical trial among 500 Black kidney transplant candidates. The main objective of this study is to test an educational and behavioral intervention that is designed to increase receipt of LDKT among transplant candidates (persons active on the deceased donor kidney transplant waiting list) who are Black. Candidates on the kidney transplant waiting list will be randomly assigned to 1 of 2 conditions: (1) a control group that will receive Usual Care, or (2) an Intervention group that will receive Destination Transplant, a 9-month intervention that includes an in-person group-based education session, postcards at monthly intervals, and a follow-up phone call from a transplant educator. At baseline and during 18 months of follow-up, demographic and clinical variables will be collected, as well as variables such as transplant derailers (factors that might be sources of delay, difficulty, or challenge to pursuing transplant), transplant knowledge, and health literacy, small steps taken to pursue LDKT, readiness for LDKT, decisional balance and self-efficacy LDKT, decisional conflict, family support, availability of potential living donors, and general health status.

**Conclusions.:**

This educational intervention aims to increase both readiness to pursue LDKT and actual receipt of LDKTs among Black and African American patients who are already on the kidney transplant waiting list. The aim of the intervention is to reduce racial disparities in access to LDKT.

## INTRODUCTION

Compared to chronic dialysis, living donor kidney transplant (LDKT) and deceased donor kidney transplant (DDKT) both offer decreased mortality, increased quality of life, and lower per person per year costs.^1-4^ LDKT, however, offers several additional advantages over DDKT. LDKT is associated with better outcomes,^5^ allows transplant candidates to bypass the long waiting list for DDKT, and minimizes dialysis duration. Therefore, for most end-stage renal disease patients, LDKT is the best treatment option for their kidney failure.

Unfortunately, Black people are much less likely than non-Black people to receive LDKTs.^6-9^ In 2018, Black people comprised 32.6% of the DDKT waiting list^10^ and comprised 32.4% of DDKT recipients but just 12.2% of LDKT recipients. The percentage of LDKT recipients who are Black has remained <15% for each year since 2000. One recent study found that all 275 transplant centers in the United States perform proportionally fewer LDKTs among Black people than non-Black people.^8^

Several interventions have been designed to help kidney transplant candidates, especially those who are Black, to learn about LDKT and identify living donors. These interventions have used videos, written materials, and in-person discussions and have targeted patients who are not yet on dialysis,^11,12^ on dialysis,^13-15^ at their initial transplant evaluation,^16,17^ and on the DDKT waiting list.^18^ Some interventions have targeted chronic kidney disease (CKD) patients early in the transplant process, before they appear for transplant evaluation.^11,13,19^

These published interventions faced many challenges to success. For example, “proximal” interventions in CKD patients early in the transplant process have had limited success in increasing LDKT rates, especially given the potentially years of time between delivery of the intervention and receipt of a LDKT.^11,13,14^ Other interventions have been underpowered, targeting CKD patients at transplant centers that usually perform few LDKTs in Black people.^16,18,20,21^ Social media apps, such as Facebook, do hold some promise. One study found that patients who used a Facebook app to post a message about their need for a transplant were 6.61 times more likely to have a donor step forward than those not using the app.^22^ Another well-studied intervention that has been shown to increase LDKT or proxies for LDKT is home visits—education delivered by health professionals in patients’ homes with their families, social network, and potential living donors present. Home visits, however, have not been widely adopted due to their cost and difficulties in implementing them.^18^ While often successful at increasing patient knowledge, positive attitudes about LDKT, and learning actions, almost all published interventions designed to increase LDKT have been unsuccessful at doing so.^23^

Patients with CKD have reported several barriers that may decrease the likelihood of receiving a LDKT.^24-28^ These barriers include lack of knowledge about LDKT,^24,27,29,30^ concerns about the donor’s future health,^24-27,30-35^ guilt and concerns about inconveniencing the living donor,^24,26,27^ difficulty in asking living donors and not knowing how to ask,^24,26,27,31,36,37^ lack of medical trust,^38-44^ and lack of interaction with recipients of successful LDKTs.^45-47^ Social, behavioral, and educational interventions to increase LDKT, especially using a health educator, would ideally address some or all of these barriers for Black patients.

Here, we describe the protocol of a randomized controlled trial that is testing the effectiveness of a multicomponent educational program called “Destination Transplant.” Our aims are to (1) examine whether Destination Transplant leads to an increase in Black people's readiness to pursue LDKT, and (2) examine the impact of the intervention on receipt of LDKTs among Black patients.

## PROTOCOL DESIGN AND METHODS

### Study Overview

We designed a parallel group, 2-arm randomized clinical trial to test the effectiveness of an educational intervention, Destination Transplant, at increasing both readiness to pursue LDKT and actual receipt of LDKT among Black transplant candidates. Before initiation of intervention activities, Destination Transplant was registered on clinicaltrials.gov (protocol no. NCT02319447) on December 18, 2014. This study was approved by the Institutional Review Boards at Saint Barnabas Medical Center (SBMC) (12-69) and Rutgers University (Pro20150001749).

### Study Population, Inclusion/Exclusion Criteria, and Randomization

#### Study Population

**FIGURE 1.**
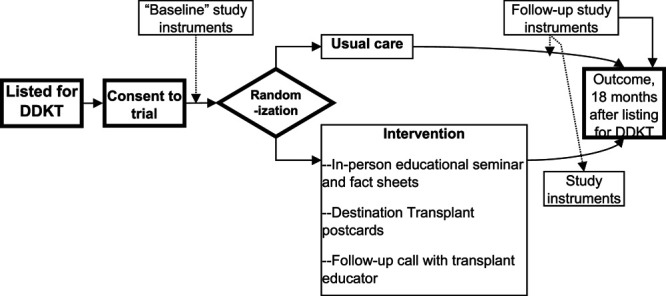
Flow of study participants through the study. DDKT, deceased donor kidney transplant.

The target population for this trial will be all kidney transplant candidates who are Black and placed on the active waiting list for DDKT at SBMC. Whenever possible, at the initial transplant evaluation at SBMC or one of our satellite locations, the study coordinator will “pre-consent” and explain the trial to potential transplant candidates who: • identify themselves as Black; • are ≥21 years of age; and • give informed consent. We will exclude potential transplant candidates who • have limited English proficiency or • are unable (eg, cognitive impairment) or unwilling to give informed consent. An overview of the study design can be found in Figure 1 below.

For the actual clinical trial, our inclusion and exclusion criteria are identical to the criteria for the preconsented group, with the following additions. The actual trial will include kidney transplant candidates who • are placed on the DDKT waiting list; and • complete the baseline questionnaire; and exclude kidney transplant candidates who • lack a working telephone; • live >150 miles from the transplant center; or • were enrolled in our prior trial of an educational intervention.^17^ Patients may elect to withdraw from study at any time. Patients who are removed from the waiting list are also withdrawn from the study.

#### Randomization

Kidney transplant candidates on the waitlist will be randomly assigned to either: (1) a control group that will receive Usual Care, or (2) an Intervention group that will receive a 9-month intervention that includes an in-person group-based education session, postcards at monthly intervals, and a follow-up phone call from a transplant educator. After completing the baseline measurement, patients who are eligible and consented to the study will be randomly assigned to 1 of the 2 conditions, in a 1:1 allocation ratio. Randomization will occur in blocks of 8, using random numbers. Randomization will be stratified by whether the study participant is newly placed on the waiting list (defined as placement on the waiting list <6 mo before enrollment) or has already been on the waiting list (defined as placement on the waiting list ≥6 mo before enrollment). Random number sequences will be generated by the offsite study biostatistician (P.O.-S.). To promote allocation concealment, the biostatistician will allocate study participants to their study arm at the time of randomization and inform the study coordinator of the assigned study arm. The biostatistician will be electronically mailed the study identification numbers of the persons awaiting randomization and will electronically mail back the allocation of each identification number to either the intervention or control groups. The study coordinator will contact potentially eligible transplant candidates by telephone to inform them of their assignment. Study participants and the study coordinator will be aware of allocation to Usual Care or the Intervention group, but outcomes assessors will be blinded to the allocation group.

### Study Aims and Objectives

The primary aim will compare LDKT readiness and LDKT receipt in the intervention versus Usual Care groups. Our primary outcome is change in readiness to pursue LDKT, and our main secondary outcome is actual receipt of LDKT after 18 months of follow-up. Other secondary aims will determine (a) the social and behavioral variables that modify the effect of the intervention upon LDKT readiness and receipt; and (b) whether the intervention affects other precursors of LDKT readiness and receipt, such as knowledge about LDKT, self-efficacy, number of donor volunteers, and other social and behavioral factors.

### Data Collection, Follow-up, and Outcomes

#### Baseline Measurements

After active placement on the waiting list, we will obtain “baseline” measurements of multiple social and behavioral attributes via telephone questionnaires of all patients enrolled in the study (see Figure 1 for flow of patients through the study). If we are unable to administer the baseline questionnaires within 2 months of enrollment into the trial (eg, within 2 months of placement on the waiting list for newly listed patients, or within 2 months of consent for the patients already on the DDKT waiting list), then we will not randomize the patient. This requirement is intended to minimize later study dropout. Study participants will be mailed a $25 gift card for this baseline measurement.

#### Follow-up Measurements: Timing

Each Usual Care study participant will be matched to 1 intervention participant. Patients randomized to the intervention will complete a follow-up questionnaire by telephone approximately 1 week after the intervention. At the same time, we will contact the matched Usual Care patient to administer the follow-up questionnaire. This procedure will ensure that the post-Intervention follow-up questionnaires are administered at approximately the same time, postlisting, for both Usual Care and Intervention patients, using the same modality (telephone). A final administration of the study questionnaires will occur 9 months after randomization for patients in both arms. We will provide $25 gift cards for each of these 2 follow-up measurements.

### Standard of Care Components (Usual Care/Control Group)

Participants who are randomized to receive the Usual Care engage in the standard education and evaluation process given to all patients at SBMC on the day of the transplant evaluation. This education and evaluation have been previously described in detail.^48^ Briefly, patients, along with any family and friends who accompany them, listen to and view a 90-minute slide presentation given by one of our trained transplant nurse coordinators. This presentation reviews topics that must be provided to potential transplant candidates, as mandated by the Centers for Medicare and Medicaid Services,^49^ including:

- treatment options for CKD;
- the evaluation process for kidney transplant;
- how the deceased donor waiting list works for kidney transplant;
- the types of LDKTs and DDKTs. These slides succinctly review the types of DDKTs, including Public Health Service, increased risk organ offers, benefits of LDKT, the workup of potential living donors, the types of living kidney donors, and alternative programs for LDKT (including paired exchange);
- the benefits and risks of kidney transplantation;
- what to expect with the surgical procedure; and
- patients’rights and responsibilities.

Transplant candidates are evaluated privately by members of the transplant team, including the transplant nurse coordinator, social worker, nephrologist, and (if needed) dietician.

While on the waiting list, transplant candidates are asked to inform the transplant center of important changes in their medical condition (eg, hospitalizations) but otherwise typically have infrequent contact with the transplant center. Candidates return to the transplant center for periodic reevaluations by transplant staff, usually every 1–2 years, depending upon the transplant candidate’s medical comorbidities. At these reevaluations, transplant personnel usually discuss the option of LDKT highlighting the possibility of receiving a transplant sooner. These Usual Care patients will receive usual concomitant care but no additional formal education. Patients are kept active on the waiting list unless there are medical or psychosocial reasons for inactivation.

### Intervention Components (“Destination Transplant”)

Destination Transplant was developed by a transplant nephrologist, a social psychologist, a health communication researcher, and a graphic designer, and is based upon the Transtheoretical Model (TTM) of Change.^50^ Destination Transplant is designed to increase both readiness to pursue LDKT and actual receipt of LDKTs among Black transplant candidates. The intervention is designed to be (1) practical (focused around a 1-time, in-person, group-based education) with additional remote contacts (follow-up phone call, monthly postcards) and therefore easily replicable if shown to be effective; (2) focused on a receptive sub-population of CKD patients—listed transplant candidates—who may be especially receptive to interventions to increase LDKT; and (3) interactive and personal, using “live” talks with actual recipients and living donors. The 9-month intervention includes an in-person group-based education seminar, postcards at monthly intervals, and a follow-up phone call from a transplant educator.

#### Destination Transplant In-person Education Seminar

The in-person education seminar is designed to provide patients with educational support and interaction with racially concordant transplant health educators and patient ambassadors. During the in-person component of the intervention, patients will engage in a single, 60- to 90-minute educational and motivational seminar, delivered to small groups of Black kidney transplant candidates and their family and friends (see Table 1 for details). The Intervention seminar will feature a slide presentation that includes brief talks from a physician, a Black patient educator and Black patient ambassadors (kidney donors and LDKT recipients). We anticipate that each seminar will include 3–5 listed transplant candidates, as well as family and friends. These seminars will be held at 1 of our 2 sites—SBMC, our main site, or our satellite location at Newark Beth Israel Medical Center. At the end of each Intervention seminar, we will measure the patients’ perceptions of the cultural competence of the physician, health educator, patient speakers.

**TABLE 1.** Intervention seminar components

Topic	Speaker
Facts about CKD, treatment options, types of DDKTs and LDKTs, and the waiting list: • basic facts about CKD • the kidneys • dialysis • benefits of transplant • the DDKT waiting list and how it works • types of DDKTs, including high KDPI kidneys • types of LDKTs, including paired donation • how to stay healthy and ready for a transplant	Transplant physicianTransplant educator
Experience of receiving a transplant and LDKT: • personal background • how the patient developed CKD • experience while on dialysis or with CKD • how they recruited a living donor • the transplant surgery and early post-transplant experience • current life with a transplant (advantages and disadvantages) • how their living kidney donor is faring after donation • misconceptions about kidney transplant • audience questions	Black LDKT recipient
Experience of serving as a live kidney donor • personal background • how the donor became aware of the need for transplant by a loved 1 • why the speaker decided to become a live donor • testing and evaluations to become a donor • the actual donation operation • life after donation; and • audience questions.	Black live kidney donor
Ways to more quickly receive a transplant • accept a high KDPI kidney, if deemed medically appropriate • accept a PHS increased risk kidney • get on the DDKT waiting list at other transplant centers outside the local donation service area • LDKT, either directly or via kidney paired donation	Transplant educatorTransplant physician
Wrap-up talk	Transplant physician

CKD, chronic kidney disease; DDKTs, deceased donor kidney transplant; KDPI, kidney donor profile index; LDKTs, living donor kidney transplant; PHS, public health service.

We developed a slide presentation as the main method of education delivery. This interactive presentation provides patients with the opportunity to learn general information about CKD, the waiting list, and different types of kidney transplant. Facts about kidney disease, the waiting list, and transplant will be presented in a question and answer format in the slide presentation. Patients will be asked to respond to true/false and multiple-choice questions regarding transplant and living donation and receive nearly immediate feedback from the education team and the physician. The presentation introduces 5 types of kidney transplants that the transplant candidates can consider:

(1) DDKT from a “standard” donor
(2) “Nonstandard”DDKT (from donors with either a high kidney donor profile index or considered Public Health Service increased risk)
(3) Multiple Listing at 2 or more transplant centers (DDKT at another transplant center in a different donation service area)
(4) Direct LDKT
(5) LDKT via kidney paired donation

The slide presentation education is designed to progress through the treatment options from least to more difficult, in terms of additional effort needed on the part of the patient. For instance, the first treatment option, DDKT from a standard donor, requires no additional action, given that each patient is already active on the DDKT waitlist. Each of the 5 transplant options will be discussed in detail, with concluding statements outlining the necessary steps for selecting each option (Table 1).

#### Destination Transplant Education Materials

Throughout the study period, patients will be given educational materials that were developed specifically for the Destination Transplant program. These education materials were modified based on early versions of stage-based, transplant education materials developed by the coinvestigator (A.D.W) for her Explore Transplant and Your Path to Transplant educational programs.

##### Destination Transplant Fact Sheets

As a supplement to the slide presentation, patients will be given a folder to take home that contains factsheets that provide an overview of the 5 types of kidney transplant. Although the content varies between the factsheets, all factsheets provide patients with instructions or further directions on what to do if they are interested in pursuing a particular option.

##### Destination Transplant Mailings

Also, during the 9-month intervention period, patients will be sent 1 educational postcard per month that serves to provide both basic information about kidney disease and additional information about the 5 treatment options. These colorful postcards feature real recipients and donors and tackle tough topics including asking someone to get tested as a donor (Table 2).

**TABLE 2.** Intervention monthly postcards

Postcard title	Postcard content
*“Your Exploration of Kidney Transplant Continues at Home”*	Welcomes patients and provides a shortlist of recommendations for patients who are waiting for a transplant
*“Consider All Types of Kidneys for Transplant”*	Encourages patients to consider nonstandard deceased donor transplant through use of High KDPI donor kidneys
*“Consider All Types of Kidneys for Transplant”*	Encourages patients to consider nonstandard deceased donor transplant through use of Public Health Service Increased Risk donor kidneys
*“Learn About Living Donation”*	Encourages patients to reach out to others for support and to consider living donor transplant as a treatment option
*“Learn Why People Want to Be Living Donors”*	Presents reasons why living donors may offer to help out a friend or a loved 1.
*“Compare the Risks and Benefits of Living Donation”*	Provides information about the risks (including costs, future impact on health, and risk of death) associated with donating a kidney
*“Consider Getting a Transplant from a Living Donor”*	Provides a list of strategies that may be used to help an individual identify a potential donor
*“You Can Get a Transplant Even if Your Donor is Not a Match for You”*	Defines and explains how Kidney Paired Donation works
*“Weigh the Pros and Cons of All Your Options”*	Provides a side by side comparison of the pros and cons of dialysis, deceased donor transplant, and living donor transplant

KDPI, kidney donor profile index.

#### 3 Months Post-baseline Follow-up Coaching Call

Approximately 3 months after the baseline assessment, patients in the intervention condition will receive a follow-up phone call from a transplant educator to discuss topics including but not limited to:

(1) their transplant plan and decision-making
(2) what supports they have and need to ensure they are able to follow through with their transplant plan
(3) how to discuss LDKT with friends and loved ones

The purpose of the call is to provide additional education and support for patients. These conversations also provide additional opportunity for patients to discuss their feelings about and willingness to pursue the various transplant options. This call serves as a mini-assessment wherein the coach assesses patient readiness to pursue LDKT. At the conclusion of the coaching call, patients are given the option to receive additional educational materials designed to help bolster their confidence in potential discussions about their need for an LDKT.

Patients expressing interest in learning more will be given the option to receive the *Explore Living Donation Packet* and the *Finding a Living Donor Booklet*. Each resource provides patients with practical, skills-based information to help initiate conversations about living donation. Through these materials, patients will engage with videos and print materials that provide detailed descriptions about how others have found living donors. Additionally, patients are given several sample letters that others have used with family and friends to help them identify potential donors.

### Measures and Variables

#### Measures

Patient information will be obtained, mainly via questionnaires, at baseline, interim follow-up (as explained above in *Follow-up Measurements: Timing*), and 9 months (Table 3). Descriptions of these measures are below. Unless otherwise indicated, all measures will be assessed at baseline, follow-up, and 9 months. Data collection forms are potentially available upon request.

**TABLE 3.** Outcomes and measures

	Baseline (after listing)	1 wk after intervention	9 mo after baseline	18 mo after baseline
**Outcomes**				
** Primary: Readiness to pursue LDKT** (stage of change)	X	X	X	
** Main secondary**: **Receipt of a LDKT**				X
** **Number of donor volunteers recruited & evaluated				X
** **Status on DDKT waiting list				X
** **Knowledge of LDKT	X	X	X	
**Other mediators, variables, and correlates**				
** Demographic and medical characteristics**	X			
** Transplant derailers**	X			
** Previous transplant education**	X			
** Health literacy**	X			
** Small steps taken to pursue LDKT**	X	X	X	
** Readiness for LDKT**	X	X	X	
** Decisional balance (pros and cons) regarding LDKT**	X	X	X	
** Self-efficacy regarding LDKT**	X	X	X	
** Family and social support**				
** Availability of potential living donors**	X			
** General health status**	X		X	
** Medical Mistrust**	X		X	
** Cultural competence**		X	X	
** Decisional conflict**		X	X	

DDKTs, deceased donor kidney transplant; LDKTs, living donor kidney transplant.

##### Demographic, Clinical, and Cultural Factors (Baseline Only)

During the baseline assessment, patients will be asked some basic demographic questions regarding factors such as their age, biological sex, race, and ethnicity. Clinical information will be collected, including data such as patient dialysis status and comorbidities such as diabetes, hypertension, and polycystic kidney disease.

##### Transplant Derailers (Baseline Only)

Transplant derailers can be described as individual factors that might be sources of delay, difficulty, or challenge to a patient pursuing transplant. These factors include education, job status, income, health insurance quality, neighborhood/environmental assessment, financial stability, access to transportation, and family obligations.

##### Previous Transplant Education and Health Literacy

To assess health literacy, the 2-item subjective health literacy and numeracy measure will be used.^51^ Extent of previous transplant education and current transplant knowledge measures were adapted from previously developed measures.^52^ The knowledge and education measures were designed to assess participants’ amount and quality of transplant knowledge.

##### Small Steps to Pursue LDKT

Small steps include a list of actions that people may take related to getting a living donor transplant (eg, talk to people you trust about whether to get a living donor transplant or ask potential donors to be tested).

##### Measures of Readiness for LDKT, Based Upon TTM

We will use previously validated scales^52^ to measure readiness to pursue LDKT and the pros and cons of living donation. The readiness measures assess how ready patients are to take actions to pursue LDKT, based upon the stages in TTM of change (eg, “I am not considering taking actions in the next 6 mo to pursue living donation” [precontemplation]). We will also measure readiness using the scales developed by Rodrigue et al,^21^ which also measure readiness for LDKT but use slightly different wording (eg, “I am not thinking about or considering live donor kidney transplantation” [precontemplation] and “I have thought about live donor kidney transplantation and I am seriously considering the possibility” [preparation]). The pros and cons assessment ask participants to rate the importance of a series of statements about transplant to their decision to pursue LDKT (eg, “I will feel guilty having someone donate to me” or “With a living donor transplant, I will be able to contribute to my family and friends sooner”).

##### LDKT Decisional Balance and Self-efficacy

Decisional balance items will measure the perceived importance to patients of the possible positive and negative outcomes of LDKT. Patients will be asked, “How important is this statement to your decision about living donor transplant?” and then be asked to respond to 12 positive and negative statements (eg, “I will feel guilty having someone donate to me,” “I will be healthier because I spent less time on dialysis”). Responses will rate the importance of each statement to their decision to pursue LDKT, measured on a 5-point Likert-type scale (1 = “Not important” to 5 = “Extremely important”). To assess LDKT self-efficacy, we will use 6 items adapted from prior studies exploring LDKT.^52^ Items will measure how confident participants are that they could continue their pursuit of LDKT even if they were faced with various challenges (eg, “You don’t know anyone who might be a living donor for you” or “You asked someone to donate and they turned you down”). Responses to these items will also be on a 5-point Likert scale (1 = “Not at all confident” to 5 = “Completely confident”).

##### Family and Social Support and Living Donor Availability

These questions measure the quality of participants’ support networks. Questions include the number of available donors, quality of the potential donors as determined by their health status, number of donor offers, and willingness to consider paired donation. Additionally, a brief assessment of Unmet Social Support Needs^53,54^ will be used that compares the amount of transplant-related support participants have received in comparison to how much they’ve needed.

##### General Health Status

The Centers for Disease Control HRQOL-456^55^ will be used as a measure of general health status. This 4-item assessment asks participants to report on overall health status (ie, physical, mental, and emotional health) in the last 30 days.

##### Medical Mistrust

Medical Mistrust will be measured using the 7-item The Medical Mistrust Index.^56^ This scale examines whether or not patients trust health organizations.

##### Cultural Competence Assessment

Participants will be asked to reflect on the cultural competence of the project staff based on in-person and telephone interactions. Items examine participant perceptions of whether staff presented clear information, treated them fairly, and were respectful and were adapted from supplemental cultural competence items that were part of the Consumer Assessment of Healthcare Providers and Systems.^57^

##### Decisional Conflict Scale (Follow-up and 9 Mo Only)

This 11-item scale asks respondents to reflect on the information they received about their treatment options and reflect on the option they chose, measured on a 5-point Likert scale of Strongly Disagree to Strongly Agree (eg, “I am clear about the best choice for me”).^58^

#### 18-Month Records Review

We will review participants’ medical records at 18 months postrandomization to capture the following transplant-related outcomes.

##### Receipt of LDKT

We will determine, via examination of medical records, whether trial participants have received a LDKT in the United States during the 18 months after they were randomized. If the person received a LDKT within the United States, then we will determine whether this LDKT occurred at SBMC or some other transplant center.

##### Recruitment of Donor Volunteers

Using electronic transplant medical records at SBMC, we will determine how many donor volunteers contacted the transplant center to donate to each study participant during the 18-month follow-up period. Persons are considered “donor volunteers” after they (1) contact the transplant program to request an information packet regarding live kidney donation and (2) complete and return a Living Donor Referral Form (included in the packet) to SBMC.

##### Evaluation (Nursing Education) of Donor Volunteers

After returning the Donor Referral Form, donor volunteers must complete an in-person education session regarding live kidney donation. Appearance at the transplant center for this education and evaluation is a sign of the “seriousness” of the donor volunteer’s intent to donate. For each study participant, we will determine how many of their donor volunteers appeared for in-person nursing education.

##### Status on the DDKT Waiting List

After the 18-month follow-up period, we will query the medical records for each study participant. For study participants still on the waiting list, we will determine whether they are • active (status 1) or • inactive (status 7) on the list. Other patients will have been removed from the SBMC waiting list because they: • died; • became too sick to transplant; • refused transplant; • transferred to another center; • improved and no longer required transplant; • received a DDKT; • received a LDKT; or • other.

### Statistical Analyses

Data will be entered electronically, stored in password-protected electronic form or locked cabinets at SBMC, and verified to be in the proper format and within expected value ranges. Distributions of baseline variables and outcomes were described using counts and percentages for categorical and ordinal variables and using means and standard deviations for continuous variables, both across the sample as well as within control and intervention arm. As this is a clinical trial, standard practice is not to create *P* values to compare significant differences in baseline information between trial arms.

Outcomes will be evaluated using standard intention-to-treat principles and recognizing the stratified randomization via time on waiting list (≤6 mo versus >6 mo). Ordinal outcomes were compared across treatment groups via the Cochran-Mantel-Haenszel statistic, a type of stratified chi-square test for binary or ordinal outcomes. Continuous outcomes will be evaluated via ANOVA (linear models). Outcomes include readiness to pursue LDKT (primary at 9 mo), receipt of LDKT at 18 months, the recruitment and evaluation of donor volunteers, change in knowledge of LDKT at 9 months, and status on the waiting list at 18 months (secondary). Secondary analyses for ordinal or binary outcomes will be stratified by time on transplant list as well as baseline level of the outcome, while linear models for continuous will control for baseline if available.

To test the effect of the intervention upon other precursors of LDKT readiness and receipt (eg, knowledge about LDKT, self-efficacy, number of donor volunteers, and other social and behavioral factors), we will use Cochran-Mantel-Haenszel tests for categorical variables and linear regression models for semi-continuous scales, stratified by accumulated time on the waitlist at randomization (≤6 mo versus >6 mo). We will examine effects adjusted and unadjusted for baseline value for each respective scale.

If there is a significant effect of intervention, we will determine which baseline factors (eg, age), if any, modify the effect of intervention on the primary and secondary outcomes using either logistic or linear regression models. In particular, we will test for significant interactions between treatment arm (intervention versus waitlist) on the potential modifier using Wald tests.

Finally, in exploratory analyses, we will use regression models to identify characteristics that are predictive of individuals who receive LDKT, regardless of treatment arm.

### Power and Sample Size Calculations

The proportion of Usual Care (control) patients who have increased readiness for LDKT could plausibly range from 0.10 to 0.40, while the proportion who receives a LDKT is expected to equal 0.14 (based on prior baseline data from our center). Assuming there are 250 or 200 (assuming 100% and 80% retention rates) participants in each group, powers for increases of 0.10, 0.15, and 0.20 are presented in Table 4. In most scenarios, we have >80% power to detect a clinically significant increase in LDKT readiness and receipt of LDKT. We may be slightly underpowered only for very small increases in these 2 outcomes.

**TABLE 4.** Power calculations for primary and main secondary outcomes

Proportion among controls	Proportion among intervention	Power	
		250 participants per arm	200 participants per arm
**Increase in readiness of at least 1 stage**			
0.10	0.20	0.85	0.76
0.10	0.30	>0.99	>0.99
0.25	0.40	0.94	0.88
0.25	0.45	>0.99	0.99
0.40	0.55	0.91	0.83
0.40	0.60	0.99	0.98
**Receipt of LDKT**			
0.14	0.24	0.78	0.68
0.14	0.29	0.98	0.95
0.14	0.34	>0.99	>0.99

LDKT, living donor kidney transplant.

### Monitoring

Given the low-risk nature of the educational intervention, no Data Safety and Monitoring Board is deemed necessary. Because the study interval is relatively short, and the risks are low, we do not plan to perform any interim analysis, and our trial will not have any stopping criteria or routine audits. Potential harms of the intervention are not expected, and patients’ status on the DDKT waiting list will be collected as an outcome.

### Dissemination

The investigators and biostatistician will have access to the full dataset. Results will be submitted to peer-reviewed journals for publication, following the authorship guidelines of the journals. Any changes or updates to the protocol will undergo the IRB review process for modifications to research protocols. Approved modifications will be shared with the research team via team meetings.

## DISCUSSION

Racial disparities in rates of LDKT have stubbornly persisted, and effective interventions to increase rates of LDKT are needed. This study will test a strategy designed to increase LDKT among patients on the kidney transplant waitlist. By intervening with motivated, listed patients and their support networks to educate them on how best to arrive at the destination of transplant, we hope to help more Black patients find living donors and receive LDKTs.

This randomized controlled trial is notable in several ways. First, we propose to intervene upon listed transplant candidates. Few interventions have targeted newly listed or listed candidates.^18^ Because these patients are medically suitable and interested in transplant, they may be more receptive to interventions about LDKT. Second, we will utilize “live” talks by actual recipients of LDKTs and live kidney donors (“patient ambassadors”). At the intervention seminars, transplant candidates and other attendees will have the opportunity to ask questions of these patient ambassadors. Such sharing and discussion may decrease Medical Mistrust among Black transplant candidates. Third, this study focuses upon kidney transplant candidates who are Black.

Our study design has several potential limitations. Our educational intervention is relatively “low-dose,” focused around a 1-time education session, with follow-up through passive education delivery and limited health educator content. It is unknown whether this intervention will be sufficient to accomplish behavior change. An intervention that includes additional education sessions over many weeks or months may have a greater chance of being effective or more effective, albeit less practical. In addition, all study participants in the intervention arm receive the same intervention. A more tailored, individualized approach could be more effective as well as satisfying for patients.^16^ Finally, interventions that utilize technology (eg, Web-based resources) may also be easier to implement and scale, especially at larger transplant programs.^59,60^

In conclusion, this study has the potential to inform clinical care and patient education in kidney transplant centers. Studies of behavioral and educational interventions implemented after placement on the waiting list for transplant might be more effective than interventions implemented earlier in the transplant process. The results of this study will enable the transplant community to determine the best ways to educate the CKD population regarding LDKT.

## ACKNOWLEDGMENTS

We would like to thank Emily Wood for her assistance with editing and article submission. We would also like to thank Explore Transplant for contribution of educational materials for this trial.
